# Multi-scale models of whole cells: progress and challenges

**DOI:** 10.3389/fcell.2023.1260507

**Published:** 2023-11-07

**Authors:** Konstantia Georgouli, Jae-Seung Yeom, Robert C. Blake, Ali Navid

**Affiliations:** ^1^ Biosciences and Biotechnology Division, Physical and Life Sciences Directorate, Lawrence Livermore National Laboratory, Livermore, CA, United States; ^2^ Center for Applied Scientific Computing, Computing Directorate, Lawrence Livermore National Laboratory, Livermore, CA, United States

**Keywords:** whole-cell modeling, systems biology, multi-scale models, data integration, high performance computing

## Abstract

Whole-cell modeling is “the ultimate goal” of computational systems biology and “a grand challenge for 21st century” (Tomita, Trends in Biotechnology, 2001, 19(6), 205–10). These complex, highly detailed models account for the activity of every molecule in a cell and serve as comprehensive knowledgebases for the modeled system. Their scope and utility far surpass those of other systems models. In fact, whole-cell models (WCMs) are an amalgam of several types of “system” models. The models are simulated using a hybrid modeling method where the appropriate mathematical methods for each biological process are used to simulate their behavior. Given the complexity of the models, the process of developing and curating these models is labor-intensive and to date only a handful of these models have been developed. While whole-cell models provide valuable and novel biological insights, and to date have identified some novel biological phenomena, their most important contribution has been to highlight the discrepancy between available data and observations that are used for the parametrization and validation of complex biological models. Another realization has been that current whole-cell modeling simulators are slow and to run models that mimic more complex (e.g., multi-cellular) biosystems, those need to be executed in an accelerated fashion on high-performance computing platforms. In this manuscript, we review the progress of whole-cell modeling to date and discuss some of the ways that they can be improved.

## 1 Introduction

Biology once was considered a data poor science. That era has long passed. Today, thanks to revolutionary advances in sequencing and other high-throughput analytical techniques, staggering amount of biological data is being collected (Marx, 2013). Soon the cost of storing and analyzing the biological data could be more concerning than the cost of generating it (Fritz et al., 2011; Berger et al., 2013; Jagadish et al., 2014; Stephens et al., 2015). Further complicating the challenge, the data that is being generated is highly heterogeneous. The data is also variable. At times, measurements from the same biosystem but from different groups, or even the same group but on different days or on different instruments could disagree with one another. Therefore, data processing and integration from widely diverse databases have become important tasks during *in silico* systematic analyses (Bajcsy et al., 2005; Shamim et al., 2010).

## 2 Whole-cell models

**FIGURE 1:**
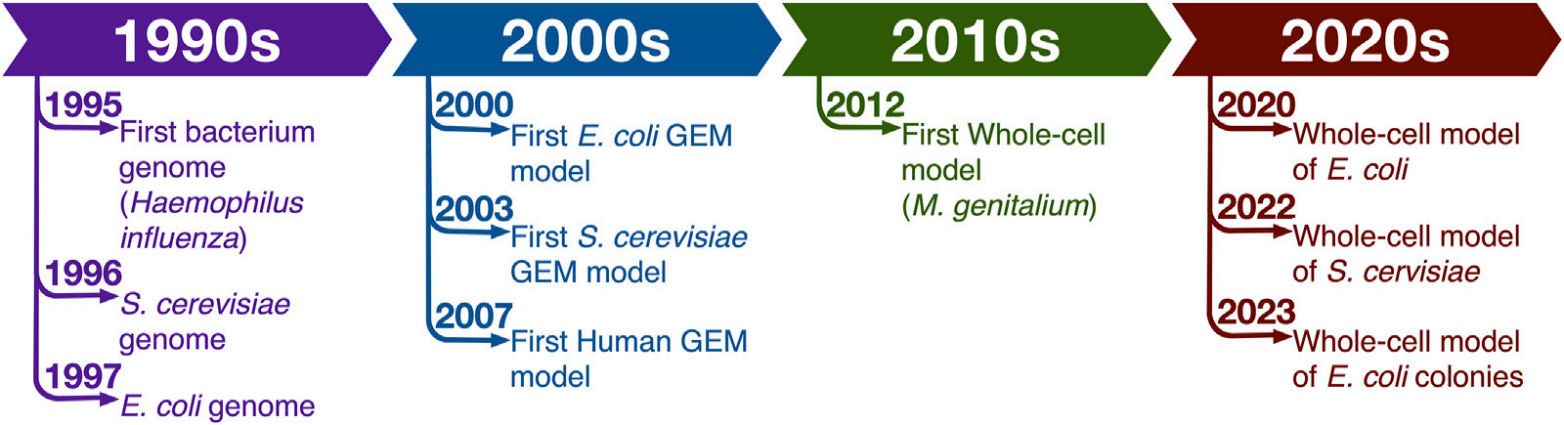
Timeline of some of the important milestones in development of whole-cell models.

French polymath René Descartes in his Discourses put forth the idea that the world behaves like a clockwork machine and therefore it can be understood by dividing it into smaller pieces and studying the individual components (Descartes, 1984). Molecular biology investigations followed this idea for most of 20th century. But while reductionist studies dominated the field and provided invaluable insights into workings of specific processes in various model organisms, the Aristotelian view that “the totality is not, as it were, a mere heap, but the whole is something besides the parts” (Cohen and Reeve, 2000) always had advocates among biologists. These detractors observed the emergent behavior of whole systems and argued that the observations that structures of systems organized and controlled the performance of the component parts refuted the reductionist basis of many studies since they failed to account for critical system-level orchestrations. For a long time, holistic analyses were impossible due to absence of system-level data. That shortcoming has now been overcome and the ready availability of various types of omics data have led to a renaissance in the field of systems biology (Figure 1).

Soon after first genomes became available, computational system-level models were developed. Genome-scale models of metabolism (GEMs) are among the most widely used system-level models. Metabolism was chosen as one of the first bioprocesses to be examined on a system-level thanks to tireless efforts of biochemists and microbiologists who for generations conducted extensive targeted mechanistic analyses of enzymes and pathways (Hill, 1970; Schilling et al., 1999; Papin et al., 2003; Cornish-Bowden, 2013; Johnson, 2013) and bioinformaticians who processed and deposited this information in numerous databases.

Coupling of GEMs with constraint-based reconstruction and analysis (COBRA) methods such as popular Flux Balance Analysis (FBA) has provided a wealth of general information regarding fundamental organization and function of metabolic pathways (e.g., (Almaas et al., 2004; Almaas et al., 2005)) while on a biosystem specific level it has shed light on the metabolic capabilities of the modeled organisms, their environmental niches and the robustness of their metabolism to environmental and genetic perturbations.

The popularity of these constraint-based modeling approaches stems from the fact that they utilize the data that is readily available (annotated genomes, empirical measurements of growth, nutrient uptake, and byproduct excretion) and circumvent the issue of dearth of kinetic data that plague generation of system-level kinetic models. Some system-level kinetic models have been developed e.g., (Klipp, 2007; Bordbar et al., 2015; Jamei, 2016), but they usually tend to account for the activity of significantly fewer genes than COBRA models due to a lack of detailed kinetic data for all cellular processes. There have been many methods developed that use Bayesian parameter estimation to predict reasonable thermodynamic and kinetic values to constrain COBRA models e.g., (Liebermeister and Klipp, 2006a; Liebermeister and Klipp, 2006b; Stanford et al., 2013) and subsequently there have been a number of attempts to add kinetic information to FBA models (e.g., (Jamshidi and Palsson, 2008; Adadi et al., 2012; Stanford et al., 2013; Chowdhury et al., 2015; Pozo et al., 2015; Khodayari and Maranas, 2016; Sánchez et al., 2017; Shameer et al., 2022)). Despite this progress, currently the vast majority of FBA models do not contain kinetic information.

Given their wide range of uses many upgrades to FBA methods have been made to incorporate heterogenous omics data into them. Many methods have been developed that constrain COBRA models with omics data other than genome (e.g., (Becker and Palsson, 2008; Chandrasekaran and Price, 2010; Zur et al., 2010; Jensen and Papin, 2011; Fang et al., 2012; Navid and Almaas, 2012; Sánchez et al., 2017; Bekiaris and Klamt, 2020; Hadadi et al., 2020; Di Filippo et al., 2022)). Several methods have also been developed that analyze multi-omics data using machine learning models prior to their incorporation into FBA models (Kim et al., 2016; Zampieri et al., 2019; Lewis and Kemp, 2021; Sahu et al., 2021). In one case, FBA was embedded into artificial neural networks resulting in a hybrid mechanistic-machine learning model that allows quantitative predictions of medium uptake fluxes based solely on medium composition (Faure et al., 2023). This development could greatly improve our ability to develop condition- and species-specific GEMs using data that are more readily available and easier to access.

There are also models available that account for the sequence-specific synthesis of gene products, their function and all catalyzed biochemical processes (Thiele et al., 2012; Ma et al., 2017). However, despite all these advances in COBRA modeling, all GEM models and upgraded variants do not fully account for activity of every known biological molecule and process. It is also important to account for the structure of the cell since most molecular processes use it to collocate into interacting modules at multiple scales (Betts and Russell, 2007). While GEMs for eukaryotes bin the reactions of metabolic reconstructions into different cellular compartments, they do not explicitly account for clustering of molecules and proteins within prokaryotes or organelles in a manner that could explain observed interacting units. Additionally, most GEMs contain many sources or sinks of energy and metabolites which hinder accurate and detailed description of mechanisms associated with homeostasis in a system (Roberts, 2014). Whole-cell models aim to overcome these limitations.

Whole-cell models, as with other “system-level” models aim to predict cellular phenotypes from genotype and biochemical and biophysical characteristics of the environment. Where WCM supersedes the other modeling efforts is the ambitious goal of incorporating the function of each gene, gene product, and metabolite in the modeled system (Karr et al., 2015). Thus, WCMs serve as nearly comprehensive knowledgebases for the modeled system. They allow *in silico* experiments that can lead to prediction of novel biological phenomena, identification of gaps in our knowledge, generation of new hypotheses and design of new studies (Tomita, 2001). The models can be easily updated with new information which can be a quick way of ascertaining the significance of new discoveries. Also, in this golden age of machine learning, regression techniques can be used to examine large heterogenous biological datasets and with a relatively high degree of accuracy predict phenotypes (Guzzetta et al., 2010; Smith et al., 2020; Guo and Li, 2023); in fact WCMs are the ideal complementary models to the black box nature of machine learning models and can provide a mechanistic underpinning to the predicted phenotypes.

### 2.1 Whole-cell model of *Mycoplasma genitalium*

The first whole-cell model, one that can reasonably claim to incorporate the activity of nearly all molecules in a system, was developed for the small bacterium *M. genitalium* (Karr et al., 2012). *M. genitalium* is a facultative anaerobic pathogen that can cause sexually transmitted diseases. In men it causes nongonococcal urethritis and in women it could cause a variety of ailments including cervicitis, endometritis, pelvic inflammation, infertility, and even unfavorable birth outcomes.

Although *M. genitalium* (MG) does have some medical significance, the main reason why it was chosen as the first organism for development of a WCM was that it has one of the smallest known genomes (∼580 kb and 480 coded proteins) (Fraser et al., 1995). Also, compared to other genomes, including well studied model organisms like *E. coli*, MG’s genome contains significantly fewer genes of unknown function. Despite its small size and complexity, the development of the MG model was still a monumental undertaking and was a very labor-intensive process. The model contains 1900 parameters from over 900 publications and is nearly 3000 pages of Matlab code. It divides the activity of all annotated MG gene products into 28 subcellular processes. To ensure the most accurate representation and simulation, the most appropriate mathematical modeling method was used for each subcellular process. To link all these disparate models together, the developers devised a hybrid modeling approach where all 28 mathematical modules are linked to a subset of other modules via 16 cell variables. Metabolism in the MG WCM uses similar metabolic reconstructions as GEMs; however, the internal fluxes of the reactions are dynamically constrained by multiplying the amount of catalyzing enzyme present in the system (a variable in the WCM) by its catalytic constant (k_cat_).

**FIGURE 2:**
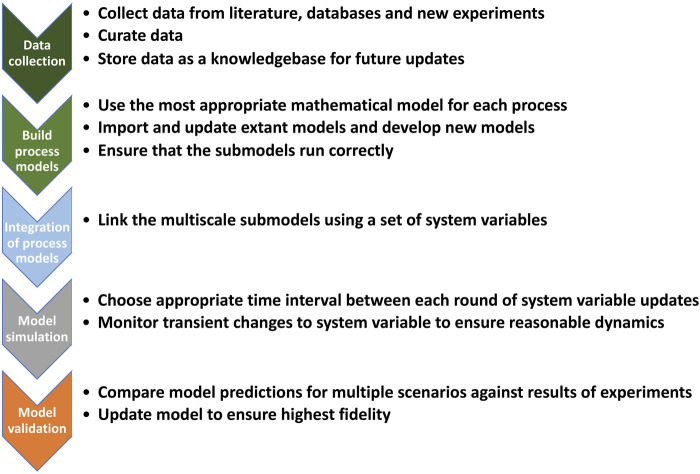
Assembly process for whole-cell models.

The simulation starts with an initial set of values for these variables. All the modules then run for a set period (e.g., 1 s) and afterwards the value of each cell variable is updated based on input from all the modules that link to it (Figure 2). Once the variables have been updated, the modules are run again using the new values. The process continues until a preset biological objective has been accomplished. Given the complexity of the problem, the amount of data that needed to be transferred back and forth between variables and modules, and the inefficiency of the solver, the simulation time for the original MG model was slow (∼1 day for 1 cell cycle). The model provided some interesting insights into working of MG and predicted some novel phenotypes.

In cases where experimental results and model predictions disagreed, gaps in our knowledge were identified and some parameter values were corrected (Karr et al., 2012). This type of model-driven knowledge gap filling and correction is a strong suit of WCMs. For example, the MG WCM was used in a follow up work by Sanghvi and coworkers (Sanghvi et al., 2013) to compare the WCM predicted growth rates for all non-lethal single-gene deletions with experimental data. In cases of quantitative disagreement between model predictions and experimental measurements, the authors examined the “molecular pathology” of each gene-deleted strain and identified gene targets which during the genome annotation process had been wrongly assigned a function or had a missing function that was not included in the model. In some other cases they identified alternate metabolic pathways that could compensate for loss of a gene product. Finally, given the more quantitative nature of WCM (in comparison to FBA models) due to their incorporation of kinetic data into their metabolic simulations; the authors were able to use the quantitative differences between model predictions and experiments to predict appropriate kinetic parameters for several critical enzymes. The predicted values were experimentally validated. Comparing the new measured values with the literature data that originally was used to train the MG WCM showed significant differences, in some cases up to four orders of magnitude.

The ability of WCMs to reliably predict in a quantitative manner the *in vivo* dynamics of a system; information that cannot easily be measured but is invaluable for assessing the state of a system and guiding efforts to alter it, makes WCMs critical tools for biological engineering projects. For example, WCMs can provide invaluable information about how incorporating synthetic gene circuits in an organism could alter the working of the system and how internal processes that are almost always unaccounted for *in silico* models can divert the system behavior away from desired outcome. In this vein, Purcell et al. (2013) used the MG WCM to examine the effects of adding genes into MG. They also examined how codon usage affects gene expression and in agreement with results from *E. coli* (Kudla et al., 2009). They found no difference in expression rates. Recently (Rees-Garbutt et al., 2020) have used the MG WCM within a design-simulate-test framework to predict a minimal genome that (if biologically correct) could be smaller than *JCVI-Syn3.0* minimal genome bacterium.

## 3 Progress

### 3.1 Whole-cell model of *Escherichia coli*

While the development of MG whole-cell model (WC-MG) was a monumental achievement and has been used to highlight the immense potential of WCM for a variety of important uses, WC-MG has limited utility for common uses of *in silico* models such as predicting targets or outcomes for bioengineering. To have that ability, the logical next organism to be modeled needed to be the best studied bioengineering chassis organism, namely, *E. coli*. To that end, a hybrid multi-math, multi-scale model for *E. coli* has been developed (WC-EC) (Macklin et al., 2020). It incorporates the function of over 40% of the well-annotated genes in *E. coli* genome (1,214 genes). Although the model does not account for activity of every gene product in *E. coli*, the model is significantly larger than the WC-MG (>10,000 mathematical equations and >19,000 parameters). This is not surprising given that *E. coli*’s genome is an order of magnitude larger than MG’s and *E. coli* has nearly 50 times more molecules. *E. coli*’s metabolism and regulatory mechanisms are also significantly more sophisticated than those for MG. Another advantage of WC-EC over WC-MG is that 100% of former’s parameters are derived from experimental measurements compared to less than 30% of the WC-MG parameters. The WC-EC, in addition to omics data, is informed by a large amount of kinetic data. This data was collected from 1,200 hand-curated papers after reviewing 12,000 papers in the BRENDA (Schomburg et al., 2002; Chang et al., 2009) database. The fact that all the parameters in WC-EC are empirically measured allowed its use for examining the cross-consistency between the disparate data sources that were used for its parameterization. The results of analyses showed that most of the data used for the development of WC-EC were consistent with predicted behaviors. However, parameter sets that were not consistent resulted in discrepancies that were alarming. For example, the incorporated data for rate of activity by ribosomes and RNA polymerases were too low to result in measured growth rates. Another interesting finding was that some essential genes are not transcribed during division cycles and yet cells proliferate. This latter finding is a strong reminder that besides the catalytic capability and concentration of an enzyme, the time course of its production and eventual degradation can also have a significant effect on the robustness of a system to environmental and genetic perturbations.

After the publication of WC-EC, its creators have initiated the *E. coli* whole-cell modeling project (Sun et al., 2021). The project aims to expand on the published WC-EC model and ultimately develop the most detailed model *E. coli* ever. The project invites input and collaboration from the scientific community to accelerate the development process. As part of this effort, updated versions of WC-EC have been developed. One update (Ahn-Horst et al., 2022) incorporates additional growth rate control regulations such as global regulator guanosine tetraphosphate, as well as dynamics of amino acid biosynthesis and translation. The additions significantly improve the WC-EC’s ability to simulate dynamics of cellular responses as a response to environmental perturbations. Another update (Choi and Covert, 2023) added accurate tRNA aminoacylation, codon-based polypeptide elongation, and N-terminal methionine cleavage mechanisms to WC-EC which permits better examination of inconsistencies between different types of measurements. The updated model was used to verify that *in vitro* tRNA aminoacylation measurements are insufficient for cellular proteome maintenance. The model predicted a positive feedback mechanism that regulates arginine synthesis.

### 3.2 Whole-cell model of Saccharomyces cerevisiae

*Saccharomyces cerevisiae*’s (SC, Brewer’s yeast) genome was the first eukaryotic genome to be sequenced (Goffeau et al., 1996). SC is an extremely important organism economically. It is genetically tractable and has been engineered through a plethora of homologous recombination techniques. Overall, SC is the best studied single cell eukaryotic organism. Given this distinction, SC was the obvious best choice for developing the first whole-cell model of a multi-compartmented organism. The yeast whole-cell model (WM_S288C) (Ye et al., 2020) was developed by expanding upon an earlier FBA model of the organism (Österlund et al., 2013). It incorporates products of 6,447 genes (100% of genome), 975 metabolites and 6,156 reactions. Overall, it includes 26 cellular processes. Unlike WC-EC, not all incorporated parameters were available from yeast experiments. So instead, measurements from other organisms were used. The WM_S288C’s predictions were validated against experimental results and when compared against predictions from its progenitor FBA model they showed significant improvement (e.g., precision of accurately predicting essential genes WM_S288C 70%, FBA model 28%). The developers used the model to conduct an extensive study of roles of various molecules in the system. They ascertained the function of 1,140 essential genes, thus providing a mechanistic understanding of vulnerable processes under different conditions. They also gained new insights into function of non-essential genes, namely, that these genes can regulate nucleotide concentrations and thus affect cellular growth rates.

### 3.3 Vivarium

As noted earlier, whole-cell models integrate a diverse set of intracellular processes using numerous simulation methods. When developing the first whole-cell model, accuracy and completeness were primary considerations. Speed of simulation was a secondary consideration. However, (Karr et al., 2012), did attempt to speed up the whole-cell simulation by executing multiple pathway sub-models simultaneously for the agreed simulation time interval using multiple CPU cores with one per pathway in Matlab (Gunawardena, 2012). This attempt exposed a few significant challenges to speeding up simulations of hybrid models. Firstly, the time interval for all pathways is restricted by the smallest time interval needed by any individual pathway. Secondly, the level of parallelism is limited by the number of pathways. Thirdly, the pathways tend to be extremely heterogeneous in terms of the computational work needed to advance within the selected time interval. Consequently, simulating the same interval for different pathways may require vastly different computing times, making the parallelization essentially ineffective.

To answer some of these problems, Vivarium (Agmon et al., 2022), a platform for integrative multi-scale modeling, has been developed. It provides an interface for combining existing models in the nested hierarchies of multiple scales via a discrete event simulation engine. This eases the software engineering task of combining smaller pathways into a larger whole-cell model. Vivarium makes it easier to combine multiple pathways together and thus allows larger models and more computational parallelism. Vivarium offers utilities to partition molecular species shared between pathways based on expected demand in such a way that mass is conserved. In this way, individual pathways can run independently from each other within a time interval. Vivarium can also leverage the message-passing of the Python multiprocessing module to exploit the inherent parallelism in the model across multiple cores and multiple processors. While the original version of Vivarium faced some of the same limitations as the original WCM models—linked timesteps, parallelism by pathways, and uneven computational load between pathways but updates have been made and are on the way that answer some of these issues (Skalnik et al., 2023).

### 3.4 Unbalanced growth and non-steady-state metabolism

In all WCMs developed so far, metabolism is solved using updated variants of FBA method that account for each enzyme’s abundance and catalytic rate constant. Typical FBA models use a rigid biomass reaction where a single set of stoichiometric coefficients define the ratio of reactants that are used for production of a set amount of biomass and a fixed set of coefficients to define the other byproducts of cell maintenance and replication (Orth et al., 2010). This balance growth assumption is valid for most conditions, particularly if one must assume a long-term analysis. However, for the development of WCMs where FBA models are integrated in a hybrid format to interact with dynamic simulations of bioprocesses with significantly shorter timescales, this assumption is problematic. To overcome this flaw, (Birch et al., 2014), developed two variations of FBA called flexible FBA (flexFBA) and time-linked FBA (tFBA) that when run simultaneously within WCMs improve the accuracy of model predictions. In flexFBA, the fixed ratios of biomass reactants have been removed in the objective function. This eliminates the classical assumption of balanced growth. In tFBA the ratios between the reactants and byproducts in the biomass equation are no longer fixed and thus the common steady-state growth constraint of classical FBA is eased. Using these methods for WCM allows for “short time” FBA which allows integration of output from different types of mathematical models.

### 3.5 Colony-scale whole system modeling

Phenotypic heterogeneity in a microbial community, particularly those that persist for more than one generation can have a significant impact resilience of a system to environmental changes and threats. Bacterial persistence, the phenomenon where genetically identical bacterial colonies behave heterogeneously to introduction of antibiotics is known to play a key role in development of antibiotic resistance in bacteria (Gefen and Balaban, 2009). The heterogenous differences could stem molecular processes, such as stochastic expression of antibiotic resistance genes (Akiyama and Kim, 2021). Mechanistic WCMs are ideal tools for gaining a system level understanding of these phenomena. But to gain a colony level perspective requires simulating many cells interacting with one another via a shared environment. Vivarium allows such multi-scale simulations and Skalnik et al. (2023) have used it to alter WC-EC model and develop the first colony level holistic model. The model was then run in parallel using cloud computing to study the emergence of antibiotic resistance in *E. coli* when treated with two antibiotics with different modes of action.

## 4 Challenges

Despite all the advances and progress in the development of WCMs over the last decades, there are still persistent fundamental challenges that hinder not only the development of new models but also any efforts to develop computational tools for accelerating model simulation. In this section, we will discuss these challenges and propose possible solutions.

### 4.1 Data collection

As the aim of WCMs is to accurately and comprehensively predict the cell behavior, a huge amount of biological data is needed for model parameterization and validation. This need increases with the complexity and size of the cell (Babtie and Stumpf, 2017). The main challenge with efforts at gathering the needed data is ensuring that the publicly available data is in a useable format. This will allow easy identification, extraction, and aggregation of high-quality data. Unfortunately, the high dimensionality, the heterogeneity, and the lack of sufficient annotation of the data pose important challenges regarding their interpretation, and reusability. These challenges have led to calls for standardization of databases, simulation softwares and overall modeling standards (Waltemath et al., 2016).

Fortunately, a variety of tools and databases have been developed to facilitate the data collection and aggregation process. These tools also ease the burden of additional curation of data. For example, there are many repositories providing pathway/genome information such as BioCyc (Karp et al., 2017), BiGG (Schellenberger et al., 2010; King et al., 2015a), WholeCellKB (Karr et al., 2013), KEGG (Kanehisa and Goto, 2000; Kanehisa et al., 2004; Kanehisa et al., 2016) and BRENDA (Schomburg et al., 2002; Chang et al., 2009). In addition, there are databases that include experimental data for a specific organism, such as EcoCyc (Keseler et al., 2011; Keseler et al., 2017) where interestingly in its latest version (Karp et al., 2023) there is a bidirectional connection with the *E. coli* whole-cell modeling project that can be used for importing data from EcoCyc to parametrize the WCM and updating the WCM with EcoCyc’s latest mechanistic information. Human curation of data collected on bioprocesses is key to developing accurate WCMs and to this end visualization of metabolic maps can provide extremely valuable insights for data integration. Network visualization tools such as Escher (King et al., 2015b; Rowe et al., 2018) and Pathview (Luo and Brouwer, 2013; Luo et al., 2017) can be used for this task. However, these tools rely on pre-drawn maps and cannot support inputs of large networks with multi-type models.

In cases when data have not been deposited in any database, literature text mining tools for extracting biological data like Integrated Network and Dynamical Reasoning Assembler (INDRA) (Gyori et al., 2017; Bachman et al., 2023), BioQRator (Kwon et al., 2014) and PubTator (Wei et al., 2013) can help with data collection and curation efforts. However, despite these resources, there are still a few problems that need to be addressed.

Some parameters still remain unknown or of poor quality. This is because while we have been generating massive amounts of omics data, we have badly neglected measuring data needed for building kinetic models. While there are databases such as BRENDA (Chang et al., 2009) that contain some kinetic parameters such as catalytic turnover rates and substrate-protein affinity coefficients, there is wide variability between measured values even for the same organisms. Sometimes, the only available data is from an organism that might be in a different phyla or even biological kingdom.

Another problem that is a major issue with all system-level biological modeling efforts is inaccurate assignment of function to gene products. It has been shown that different annotation tools can assign widely different functions for the same proteins, particularly for proteins of non-model organism (Griesemer et al., 2018). WCMs’ ability to reconcile kinetic parameters is another significant means in our toolbox for overcoming the errors prevalent in the data we use for model parameterization. Given that WCMs integrate large heterogenous sets of data, they can be used to examine the incorporated data and through cross-validation improve the accuracy of model parameters. These types of data cross-validation and correction have already been shown to be a strength of WCMs (Sanghvi et al., 2013; Macklin et al., 2020).

Finally, we have been mostly overlooking the activities of “underground” metabolic processes in our models. Underground metabolic processes are biochemical reactions that occur due to promiscuity of enzymes. In our biological network reconstructions, we usually only include the canonical function for a protein and associated reactions if the proteins are enzymes. We typically ignore low flux reactions that occur when proteins interact with alternate metabolites. While the activity of underground metabolism under most conditions is very low, under extraordinary conditions their reaction rates can significantly increase and lead to evolution of new pathways and adaptation to new environments (Notebaart et al., 2018). Omission of underground metabolic processes from WCMs could affect the accuracy of model predictions, particularly when examining the behavior of a system under stress.

A promising solution to the problem of poor quality or missing parameters can be use of sophisticated machine learning techniques. Using big biological datasets with state-of-the-art methods like deep learning approach for symbolic regression (Petersen et al., 2019), where interpretable models can be generated by inferencing the optimal format of equations and parameters from given data, could predict some of these values.

### 4.2 Data and model integration

Combining heterogenous data together is a labor-intensive process, though advances are being made that make it easier to use disparate data and assemble it into a large model. The biomodels database (Juty et al., 2015; Malik-Sheriff et al., 2020) is one such database that captures reaction and metabolic pathways for many different cellular models. The model physiome project (Hunter et al., 2006) offers another. An ideal way of accelerating the process of WCM development is to import extant models and use them as submodels in WCMs. Chelliah et al. (2015) and Pan et al. (2021) have offered means to automatically and programmatically link disparate submodels together into one cohesive whole. Bouhaddou et al. (2018) make the case that it is important to distribute the tools and thus conditions needed for a study can be “unit-tested” like software subroutines. In this way each individual model can be checked for errors and results can be reproduced in isolation before assembled into a larger whole. Other groups agree about the need for greater reproducibility for computational models (Papin et al., 2020; Niarakis et al., 2022). Developments of tools like Memote (Lieven et al., 2020) for standardizing the GEMs and FROG ensemble of analyses for ensuring reproducibility of published models (Tatka et al., 2023) have significantly increased confidence in the quality of models that will be incorporated in future WCMs.

Though advances are being made in automatically assembling disparate data together, researchers must take care to make sure each data source is appropriate for the task at hand. This requires an extensive literature search with proper data provenance to ensure each pathway and parameter is appropriately sourced and justified.

Once this data is assembled, deciding how best to simulate the model is no small task. From a software engineering standpoint, reference code implementations from different research teams are usually completely incompatible with each other. This requires recoding and translating, which is why having reproducible results are so important. Model definition languages like SBML (Hucka et al., 2018), CellML (Lloyd et al., 2004), and Modelica (Fritzson and Engelson, 1998) offer an advantage here because they separate the model definition from its numerical implementation, which simplifies composing different cellular models from different sources.

From a mathematical/numerical analysis standpoint, it can be difficult to decide how to integrate the different models into one cohesive whole that can offer numerically sound predictions. How the hybrid modeling process deals with the different time scales for the various types of mathematical models is a major challenge. For example, FBA models do not follow a time-varying process at all—they assume that the system operates at steady state and instantaneously adjusts to changes in order to optimize some biological objective. Ordinary differential equations (ODEs) and stochastic differential equations (SDEs) give continuous approximations of the evolution of high-concentration chemical concentrations within a component. There are well-established best practices on how to simulate ODEs/SDEs accurately, but best practices like simulating all the equations together with a global adaptive timestep fall at odds with WCM’s practical need to modularize and separate different subcomponents from each other. For low-concentration chemical pathways, simulation methods like discrete chemical kinetics are preferred (Gillespie et al., 2007; Gillespie et al., 2013). Putting these disparate mathematical models together is hard, and care must be taken to ensure that artificial numerical artifacts are not introduced in the process. Here are some examples of difficulties that can arise when combining multiple different mathematical models.

• Each numerical method has different time stepping requirements. It is unclear how one determines which method controls the global timestep.
• The frequency of synchronization between different numerical mathematical models is unknown.
• In cases when ODE method is extremely stiff and requires miniscule timesteps the simulation can grind to a halt.
• The method for synchronizing continuous models like ODE/SDE with discrete chemical kinetics is unknown.
• When the concentration of a molecule gets too low in an ODE model there is a need to switch to discrete chemical kinetics. Current hybrid modeling method cannot handle this switch.
• At times it will be necessary for models to evolve independently from each other while at other times they need to be tightly coupled and must be solved together. This requires an evolving architecture of links between submodels and system variables which currently is unavailable.

None of these problems have simple solutions. It is up to the individual research teams to find the modeling format that provides the most accurate predictions and useable models. However, this level of variance could drastically lower the reusability of the models for other studies.

Aside from physical and mathematical scaling problems, from a computational viewpoint, solving the different types of models can be quite intense. FBA simulators require linear programming solvers, which have O(n^3^) computational requirements (i.e., every time the size of the model doubles, you need eight times the computational resources). As models get larger, it is unclear how one can spread this work across many processors to speed up the simulation. ODE/SDE solvers are usually extremely efficient, but whole-cell modeling is an inherently multi-physics and multiscale problem, with stiff processes that evolve/oscillate on a microscale timescale interacting with processes that evolve on a timescale of days. How do you synchronize these disparate timescales efficiently, and how do you separate the workflow onto multiple processors without incurring too much communication overhead? Discrete chemical kinetics require timing and tracking every chemical reaction in a cell. As concentration increases, your timestep becomes prohibitively small. How do you keep these systems from dominating the computational running time as they interact with high-concentration ODE models? How do you split these discrete chemical reactions onto multiple processors to help distribute the computational load?

### 4.3 Slow simulators

Although development of Vivarium (Agmon et al., 2022) has helped with some of the issues that plague simulation speed of complex whole-cell models, it is still limited to running on a single CPU with multiple cores although in principle it can extend to support distributed memory systems. Nevertheless, load balancing remains challenging while limiting the speedup.

While it might be possible to answer some of the problems associated with simulation of complex systems by building accurate reduced models (e.g., (Gates et al., 2021; Avanzini et al., 2023)), alternative solutions have been proposed. Goldberg et al. (2016) envision highly parallel whole-cell simulations by clustering species and reactions into groups that interact infrequently with each other and by simulating them in the parallel discrete event simulation (PDES) paradigm (Jefferson et al., 1987). PDES enables further parallelism otherwise difficult to leverage via speculative execution and rollback management (Jefferson et al., 1987). This requires elaborate implementation and is currently under development.

Other potential remedies include parallelization of individual sub-models, especially the computationally demanding ones. Among the modeling approaches used in whole-cell models, stochastic simulation algorithm (SSA) (Gillespie, 1976; Gillespie, 1977) implements the most detailed model of discrete biochemical reaction events. SSA is necessary for accurately simulating statistically correct trajectories of species especially with low constituent counts. As more and more kinetic data become available for developing more accurate models, SSA can be used to simulate larger reaction networks. However, its computational cost is prohibitive for the scale of whole-cell models, even for the smallest organisms.

A popular approach to speed up an SSA simulations is to simultaneously execute multiple independent realizations of a simulation (Klingbeil et al., 2011; Sanft et al., 2011). Unfortunately, this approach is not directly beneficial to whole-cell modeling as it couples SSA-based models with other types of models for a simulation run.

However, there exist a variety of SSA methods (Gillespie, 1977). Especially, the next reaction method (NRM) (Gibson and Bruck, 2000) exposes opportunities for parallel processing. It employs a dependency graph to identify the coupling between reactions via their commonly referenced species (biomolecules in WCMs), and to selectively update the propensity and the time of the next occurrence of each reaction impacted by the fired one (Gibson and Bruck, 2000). Such updates can be processed independently of each other (Yeom et al., 2021). The degree of parallelism here is bounded by the number of system updates, i.e., the number of reactions involving the species consumed or produced by the reaction fired as well as the cost reduction in updating the priority queue. Some species may be shared by many reactions. This will result in a non-trivial number of updates, exposing the performance optimization opportunity. Goldberg et al. theorizes a PDES-based approach to parallelize SSA for distributed memory systems (Goldberg et al., 2020).

The cost of a single update itself may not be significant and dedicating a processor to that may not be beneficial. Therefore, an existing approach partitions the reaction network into multiple subnetworks and updates them simultaneously with one processor per group of reactions of each subnetwork via OpenMP (Yeom et al., 2021). Partitioning a network of highly skewed degree distribution for load balancing is known to be challenging (Gonzalez et al., 2012; Yeom et al., 2014). In the bipartite-graph abstraction of biochemical networks, a reaction node represents a computation, and a species node does a state. The edge indicates the dependency of the computation on the states. If a state is referenced by different reactions across multiple subnetworks over distributed memory systems, state replication, maintained by a means of coherent updates, may help mitigate the message passing cost. When parallelized for shared memory systems, the state must be accessed in a coordinated fashion among different processors to maintain consistency (Yeom et al., 2021). For balancing compute loads across processors, partitioning must consider the distribution of aggregate reaction update rates of subnetworks, which dynamically evolve through the course of simulation. This presents another challenge for load balancing and may require re-partitioning.

There exist works that parallelize SSA using accelerator hardware (Indurkhya and Beal, 2010; Komarov and D'Souza, 2012; Manolakos and Kouskoumvekakis, 2017). However, these approaches assume only the mass-action type reactions (van der Schaft et al., 2013) and leverage it for parallelization. These do not support general forms of reaction rate formula to accommodate diverse modeling practices in the field, or do not support the community standard model description, such as SBML, to its full reaction expression capacity (Bornstein et al., 2008; Sayikli and Bagci, 2011; Erdem et al., 2022).

ODE is another common simulation method used in WCM, and there exist solver packages that speed up by distributed memory parallelism using MPI along with node-level acceleration using GPU or OpenMP (Fidler et al., 2019; Balos et al., 2021; Städter et al., 2021; Elrod et al., 2022).

## 5 Conclusion

The field of whole-cell modeling is growing. Since the publication of the first WCM a decade ago a handful of models for important research, industrial, and medicinal model systems have been developed. Other than the ones mentioned above earlier, WCMs have been developed for JCVI-syn3A (Thornburg et al., 2022) and human epithelial cells (Ghaemi et al., 2020). Given the difficult and very labor-intensive process of developing WCMs, this is a remarkable achievement and a testament to how scientists view the potential of these models. The creation of these models has led to the development of whole-cell structural models (Maritan et al., 2022; Stevens et al., 2023) and even multicellular whole community models (Skalnik et al., 2023).

There are still several problems that need to be addressed before the use of these models becomes as common as usage of genome-scale models of metabolism. These include problems with data collection, model integration and parallel simulation of hybrid models. However, advances thus far are a good indication that these obstacles will soon be overcome.
